# A Tale of Three Species: Adaptation of Sodalis glossinidius to Tsetse Biology, *Wigglesworthia* Metabolism, and Host Diet

**DOI:** 10.1128/mBio.02106-18

**Published:** 2019-01-02

**Authors:** Rebecca J. Hall, Lindsey A. Flanagan, Michael J. Bottery, Vicki Springthorpe, Stephen Thorpe, Alistair C. Darby, A. Jamie Wood, Gavin H. Thomas

**Affiliations:** a Department of Biology, University of York, York, United Kingdom; b University of Liverpool, Institute of Integrative Biology, Liverpool, United Kingdom; c Department of Mathematics, University of York, York, United Kingdom; Georgia Institute of Technology School of Biological Sciences

**Keywords:** metabolism, microbiome, physiology, symbiosis, vector biology

## Abstract

The tsetse fly is the insect vector for the Trypanosoma brucei parasite, the causative agent of human African trypanosomiasis. The colonization and spread of the trypanosome correlate positively with the presence of a secondary symbiotic bacterium, Sodalis glossinidius. The metabolic requirements and interactions of the bacterium with its host are poorly understood, and herein we describe a metabolic model of S. glossinidius metabolism. The model enabled the design and experimental verification of a defined medium that supports *S. glossinidius* growth *ex vivo*. This has been used subsequently to analyze *in vitro* aspects of *S. glossinidius* metabolism, revealing multiple unique adaptations of the symbiont to its environment. Continued dependence on a sugar, and the importance of the chitin monomer *N-*acetyl-d-glucosamine as a carbon and energy source, suggests adaptation to host-derived molecules. Adaptation to the amino acid-rich blood diet is revealed by a strong dependence on l-glutamate as a source of carbon and nitrogen and by the ability to rescue a predicted l-arginine auxotrophy. Finally, the selective loss of thiamine biosynthesis, a vitamin provided to the host by the primary symbiont Wigglesworthia glossinidia, reveals an intersymbiont dependence. The reductive evolution of *S. glossinidius* to exploit environmentally derived metabolites has resulted in multiple weaknesses in the metabolic network. These weaknesses may become targets for reagents that inhibit *S. glossinidius* growth and aid the reduction of trypanosomal transmission.

## Abstract (precis)

Human African trypanosomiasis is caused by the Trypanosoma brucei parasite. The tsetse fly vector is of interest for its potential to prevent disease spread, as it is essential for T. brucei life cycle progression and transmission. The tsetse’s mutualistic endosymbiont Sodalis glossinidius has a link to trypanosome establishment, providing a disease control target. Here, we describe a new, experimentally verified model of S. glossinidius metabolism. This model has enabled the development of a defined growth medium that was used successfully to test aspects of *S. glossinidius* metabolism. We present *S. glossinidius* as uniquely adapted to life in the tsetse, through its reliance on the blood diet and host-derived sugars. Additionally, *S. glossinidius* has adapted to the tsetse’s obligate symbiont Wigglesworthia glossinidia by scavenging a vitamin it produces for the insect. This work highlights the use of metabolic modeling to design defined growth media for symbiotic bacteria and may provide novel inhibitory targets to block trypanosome transmission.

## INTRODUCTION

It has been estimated that only 1% of all microbial life is culturable (1–3). Included in this are a vast array of symbiotic bacteria. Interspecies competition, as well as sensitivity to temperature and pH and availability of oxygen and nutrients, means many species cannot be cultured using standard conditions (3–5). The ability to culture medically significant microorganisms is an important tool in disease control. Medically significant microorganisms include pathogens and the key symbionts within the system. Improved culture methods, combining microbiology with genomics, have been used to analyze the microbial flora of a number of disease vectors. Notable examples are members of the family *Paenibacillaceae* and Serratia marcescens in the Asian malarial vector Anopheles stephensi (6), the more-complex flora of Aedes aegypti (7), and the defined microbiome of the tsetse fly, the insect vector for the Trypanosoma brucei parasites that cause human African trypanosomiasis (HAT) (8).

HAT is endemic in 36 countries in sub-Saharan Africa, with an estimated 65 million people at risk of infection (9–11). The tsetse, genus *Glossina*, also hosts a limited bacterial microbiome alongside the parasitic T. brucei. The microbiome consists of a primary, obligate symbiont Wigglesworthia glossinidia and typically, a secondary facultative symbiont Sodalis glossinidius (12, 13). S. glossinidius is of medical importance, as its presence correlates positively with the ability of the tsetse to be infected by T. brucei (14–17). Its complement of more than 1,500 pseudogenes and its large genome size of 4.17 Mb are consistent with it making a rapid and recent movement from free-living to a host-restricted niche (18, 19). The high rate of pseudogene accumulation is consistent with the loss of many cellular processes and metabolic pathways that are no longer needed for life in the tsetse. These include genes involved in the transport of carbohydrates not present in the blood meal (18) and of l-arginine biosynthesis (19). The recent discovery of a closely related, free-living species of *Sodalis*, Sodalis praecaptivus, provides a useful, relevant comparison (20). It enables informed predictions to be made about the presence or absence of key metabolic genes in *S. glossinidius*. The hypothesis that *S. glossinidius* has specifically lost metabolic capabilities during its transition to symbiosis can also be tested.

Symbiotic bacteria often present with small, degraded genomes (21). As a result of gene loss and inactivation, symbionts often cannot be grown outside their host. *S. glossinidius* can be cultured, but it requires undefined rich media (13) and a longer incubation time than that for the free-living S. praecaptivus (20). This increases the risk of contamination by faster-growing organisms and limits *in vitro* study of metabolite essentiality. A rationally designed growth medium was achieved in a landmark paper for the causative agent of Whipple’s disease Tropheryma whipplei (22), but this medium still contained undefined components. An entirely defined medium will improve the culturing of *S. glossinidius* and the study of its physiology dramatically. This may then enable genetic manipulation of this organism to express antiparasitic molecules toward the elimination of T. brucei (23–25). This process is already a consideration for the control of other vector-borne diseases (26, 27).

To define an *S. glossinidius*-specific growth medium, with the eventual aim of understanding more about the symbiont’s biology and metabolic dependencies, an experimental approach was combined with whole-genome metabolic modeling (GEM) and flux balance analysis (FBA) to model *S. glossinidius in silico*. This is a powerful method when based on a well-annotated genome and the ability to test *in silico* hypotheses experimentally (28). Analysis of the metabolic network of *S. glossinidius* was first undertaken by Belda et al. (29), who described a network of 458 gene products and 560 reactions, *i*EB458. A key finding was the pseudogenization of the phosphoenolpyruvate (PEP) carboxylase gene (*ppc*), preventing the conversion of PEP to oxaloacetate for the tricarboxylic acid (TCA) cycle. The pseudogenization of components of the l-arginine biosynthesis pathway indicate the requirement of an external source of l-arginine to supplement growth *in silico*. They concluded that exogenous l-arginine is required both as a biomass component and to form succinate via putrescine in order to supplement the TCA cycle in the absence of *ppc*. The common hexose sugar d-glucose is given as the sole carbon source. Importantly, this construction of *i*EB458 was limited by the lack of a well-annotated relative from the same genus, which is no longer an issue since the discovery of *S. praecaptivus*.

We present here a significantly advanced and improved model, *i*LF517, and describe how it has enabled the development of an entirely defined medium that supports *S. glossinidius* growth *in vitro* (SGM11). Our data indicate the use of a carbon source lacking in the blood meal, namely, *N*-acetyl-d-glucosamine (GlcNAc). This suggests a complex nutritional interaction of *S. glossinidius* with the tsetse chitinous peritrophic matrix. Degradation of this by a microbe-derived chitinase might explain the increased persistence of the trypanosome when *S. glossinidius* is present (14, 15). Using SGM11, we demonstrate that *S. glossinidius* is not, as thought previously, a true auxotroph for l-arginine. Rather, it has a unique vitamin auxotrophy for thiamine, likely provided by the primary symbiont *W. glossinidia* (30, 31), through an interaction currently undefined.

## RESULTS

### The genome of *S. praecaptivus* enables an improved analysis of the *S. glossinidius* metabolic network.

*S. praecaptivus* is the only free-living member of the *Sodalis* genus to have been characterized. It has a 5.16-Mb genome with a 57.5% GC content (20). Using this discovery, S. praecaptivus was compared to *S. glossinidius* to reassess the existing metabolic model of the symbiont. This additional information verified many of the important findings in *i*EB458, while others relating to carbon and nitrogen usage were not supported.

**FIG 1:**
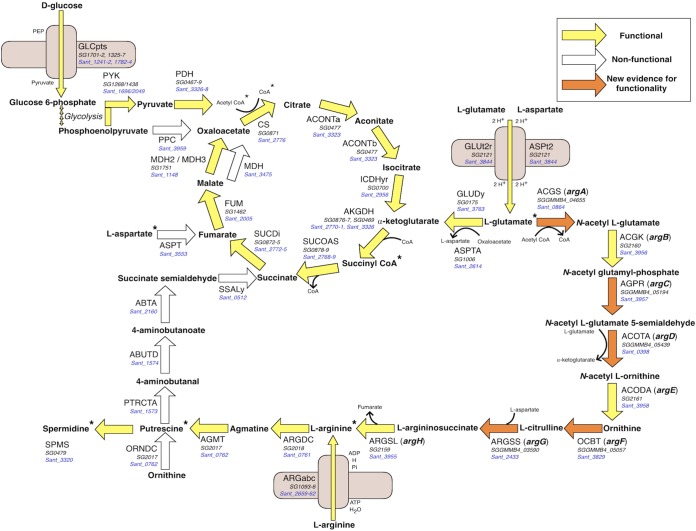
Overview of central metabolism in the *S. glossinidius* metabolic network. Functional (yellow) and nonfunctional (white) pathway components are indicated, with the *S. glossinidius* and *S. praecaptivus* gene associations given in black and blue type, respectively. Reactions for which new evidence has suggested that they might be functional are given in orange. The TCA cycle is complete, and transporters for l-arginine, l-glutamate, and l-aspartate are present. PPC and the reactions that connected ornithine with the TCA cycle are pseudogenized. There is evidence from the new genome annotation that the l-arginine biosynthesis pathway from l-glutamate may not be entirely pseudogenized, as previously thought (27). Selected coreactants are included. Asterisks indicate biomass components.

One central hypothesis derived from *i*EB458 comes as a consequence of the inactivation of the PEP carboxylase reaction encoded by *ppc* (29). This loss in *S. glossinidius* should pose a problem for its metabolism, as it loses a route to replenish oxaloacetate from PEP. This represents an important anapleurotic reaction to maintain high flux through the TCA cycle in the related bacterium Escherichia coli. To compensate for this loss, Belda et al. (29) hypothesized a threefold function for exogenous l-arginine for *S. glossinidius*: as a biomass component, as a biosynthetic precursor to putrescine and spermidine, and as an anapleurotic substrate via succinate (Fig. 1). A functional *ppc* gene is present in *S. praecaptivus* (*Sant_3959*), whereas the gene in *S. glossinidius* contains multiple frameshifts and premature stop codons (see Fig. S1 in the supplemental material), suggesting loss as a result of selection pressures or genetic drift.

10.1128/mBio.02106-18.1 FIG S1 Analysis of a portion of the *S. glossinidius ppc* gene in Artemis. Intact coding sequences are indicated in yellow. Vertical lines represent stop codons. The *ppc* gene (dark gray) is split across three reading frames due to frameshift mutations and contains two premature stop codons. Download FIG S1, TIF file, 4.4 MB. Copyright © 2019 Hall et al. 2019 Hall et al. This content is distributed under the terms of the Creative Commons Attribution 4.0 International license.

The proposed anapleurotic link from l-arginine to succinate is not supported in our analysis, with little or no evidence for these genes being present in *S. glossinidius* (Fig. 1; see also Data Set S1 in the supplemental material). l-Arginine must link to the TCA cycle to serve as an anapleurotic substrate. In *i*EB458, this linkage was proposed to occur via putrescine transaminase (PTRCTA), aminobutyraldehyde dehydrogenase (ABUTD) and 4-aminobutyrate transaminase (ABTA) (29) (Data Set S1). PatA is required for this PTRCTA reaction, but BLASTp searches find no evidence of an orthologue in *S. glossinidius* (Fig. 1). There is, however, a functional *patA* gene in *S. praecaptivus* (*Sant_1573*). Similarly, there is no functional orthologue of *patD* for ABUTD, nor of *puuE* or *gabT* for ABTA. l-Arginine can still be converted to agmatine and then to putrescine via arginine decarboxylase (ARGDC, SG2018) and agmatinase (AGMT, SG2017). This is the only route to the synthesis of this biomass component, meaning a source of l-arginine in the cell is still predicted to be essential. However, it is not supporting the proposed additional anapleurotic function.

10.1128/mBio.02106-18.7 DATA SET S1 Metabolic model of *i*LF517 for *Sodalis glossinidius*. Compounds are listed in the first tab, reactions in the second tab, and the biomass composition in the third tab. Download Data Set S1, XLSX file, 0.1 MB. Copyright © 2019 Hall et al. 2019 Hall et al. This content is distributed under the terms of the Creative Commons Attribution 4.0 International license.

An alternative organic compound must therefore serve the role of supplying TCA cycle intermediates in the absence of *ppc*. Removal of the ABTA reaction from *i*EB458, breaking the link of putrescine to succinate, results in zero biomass production. This can be rescued by the *in silico* addition of metabolites that can be introduced easily into the TCA cycle: the amino acid l-aspartate or l-glutamate or the organic acid succinate, fumarate, or α-ketoglutarate. The loss of *ppc* therefore requires the addition of a second organic substrate in addition to glucose for growth, suggesting an adaptation to an amino acid-rich environment that results from the tsetse blood diet. Important components, including l-aspartate and l-glutamate, are predicted to be present at high concentrations.

### A revised metabolic model, *i*LF517, for *S. glossinidius*.

A systematic reanalysis of the *S. glossinidius* genome enabled the construction of an independent metabolic model, *i*LF517. This model has significant differences to *i*EB458 (29). Growth was not supported in *i*LF517 using the uptake of oxygen, d-glucose, and l-arginine given in *i*EB458, indicating that alternative carbon and nitrogen sources are used. *S. praecaptivus* was used as a comparator to assess the presence of important metabolic genes in *S. glossinidius*, a resource not available to Belda et al. (29). Full details of all reactions removed from *i*EB458 and those added to *i*LF517 are highlighted in Data Set S1. *i*LF517 contains 517 genes, 703 metabolites, and 638 reactions (excluding pseudoreactions). This model can be viewed and analyzed through a web-based FBA browser on DETOXbase (www.detoxbase.org/publications/iLF517).

*i*LF517 was analyzed via FBA to investigate metabolite essentiality and the presence of predicted auxotrophies. Eighty reactions are included in *i*LF517 that were absent in *i*EB458, and 32 have been removed (Data Set S1). *i*EB458 simulates high oxygen transfer rates, using an uptake value of 20 mmol g DW^−1^ h^−1^ (DW stands for dry weight). This value was selected originally for E. coli and indicates highly aerated growth conditions in a chemostat (32–34). However, *S. glossinidius* is sensitive to high levels of oxygen (13). Cultures used here were grown in conditions of reduced aeration in comparison to E. coli or *S. praecaptivus*. The oxygen uptake rates given in *i*EB458 are therefore unrealistic for the simulations. Decreasing the oxygen supplied to *i*EB458 *in silico* results in a decrease in biomass output (Fig. S2), demonstrating that using unrealistic oxygen uptake rates exaggerate possible growth. The oxygen uptake rate in *i*LF517 was subsequently reduced to 12 mmol g DW^−1^ h^−1^, guided by the value given in a model of another microaerophile, Helicobacter pylori (35).

10.1128/mBio.02106-18.2 FIG S2 Change in biomass output with increasing oxygen in *i*EB458. Supplemented oxygen was increased until a plateau in biomass output was observed. *i*EB458 is supplemented with 20 mmol of oxygen g DW^−1^ h^−1^. This is reduced to 12 mmol DW^−1^ h^−1^ in *i*LF517 in line with the microaerophilic H. pylori. Download FIG S2, TIF file, 5.5 MB. Copyright © 2019 Hall et al. 2019 Hall et al. This content is distributed under the terms of the Creative Commons Attribution 4.0 International license.

### A defined medium, SGM11, supports *S. glossinidius* growth.

The use of complex media is insightful for examining certain aspects of bacterial physiology. However, it does limit the ability to investigate all metabolic functions. A defined medium with components of known concentrations is therefore desirable. *i*LF517 was used to design a defined medium, SGM11, containing metabolites that the model predicts may enhance *S. glossinidius* growth or become limiting.

**FIG 2:**
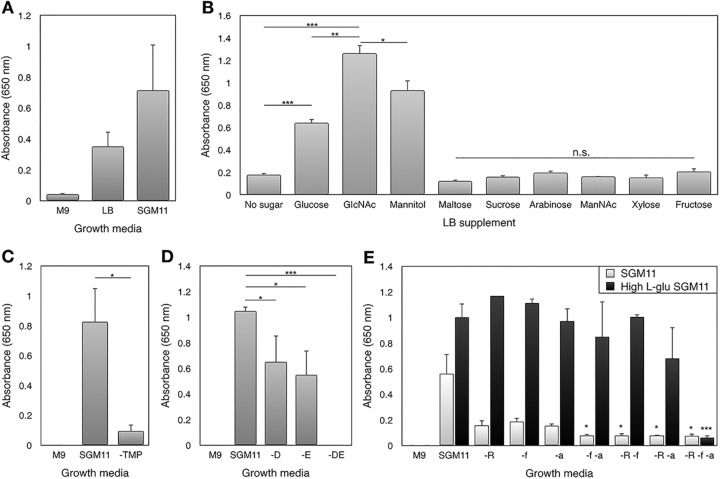
Testing *S. glossinidius* metabolism and *i*LF517 predictions experimentally. (A) The custom, defined growth medium SGM11 supports *S. glossinidius* growth to an average optical density at 650 nm of approximately 0.7. (B) Supplementation of LB with d-glucose, GlcNAc, or mannitol results in significantly greater *S. glossinidius* growth than without supplementation of a carbon source. No other carbon source had a significant improvement. GlcNAc supplementation results in significantly greater growth in comparison to either d-glucose or mannitol. The no-sugar treatment represents pooled triplicates from two experiments; the mannitol treatment represents five replicates pooled from two experiments. (C) *S. glossinidius* cannot grow in SGM11 when thiamine monophosphate has been removed (-TMP). (D) The removal of l-aspartate (-D) or l-glutamate (-E) from SGM11 reduces *S. glossinidius* growth significantly (*P* < 0.05). Removing both (-DE) abolishes growth entirely (*P* < 0.001). (E) Removing l-arginine (-R), fumarate (-f), or α-ketoglutarate (-a) from SGM11 (light gray) impairs *S. glossinidius* growth. Removing two or all of these metabolites reduces growth significantly (*P* < 0.05). In SGM11 with high l-glutamate (black), only one of these metabolites is required to support normal growth. Removal of l-arginine, fumarate, and α-ketoglutarate abolishes growth (*P* < 0.001). Measurements show the endpoint growth in triplicate, unless specified. Error bars show standard errors of the means (SEM). Values that are significantly different by one-way ANOVA are indicated by bars and asterisks as follows: *, *P* < 0.05; **, *P* < 0.01; ***, *P* < 0.001. Values that are not significantly different (n.s.) are indicated.

No growth was observed after 72-h incubation in M9 medium (36) supplemented with GlcNAc as a carbon source (Fig. 2A). The addition of trehalose, l-serine, l-arginine, l-proline, l-glutamate, l-aspartate, nicotinamide, α-ketoglutarate, fumarate, and thiamine monophosphate (TMP), to create SGM11, resulted in higher yields than with LB alone, although this difference was not statistically significant (Fig. 2A). Cell concentration was estimated using flow cytometry as approximately 3.8 × 10^8^ (standard error of the mean [SEM], 9.7 × 10^7^) for an OD_650_ value of 0.28 (Fig. S3) in order to gain an indication of the number of cells that growth in LB corresponds to. SGM11 provides a defined starting point to test the essentiality of key metabolites.

10.1128/mBio.02106-18.3 FIG S3 Flow cytometer gating for DAPI-stained *S. glossinidius* cell count. Download FIG S3, TIF file, 3.8 MB. Copyright © 2019 Hall et al. 2019 Hall et al. This content is distributed under the terms of the Creative Commons Attribution 4.0 International license.

### *S. glossinidius* maintains a reliance on a sugar, namely, the host-derived *N*-acetyl-d-glucosamine.

**FIG 3:**
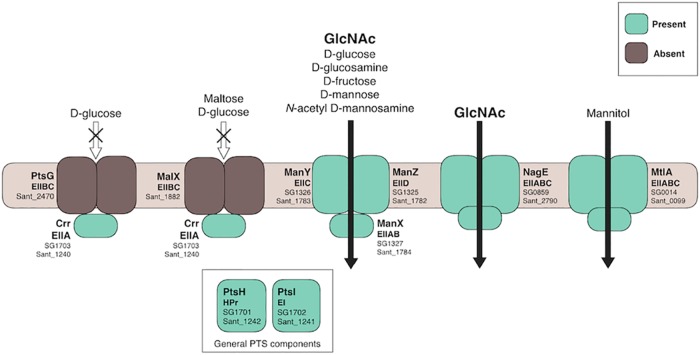
Predicted PTS transport in *Sodalis* species. The presence (green) or absence (brown) of PTS proteins in *S. glossinidius* is shown, with the corresponding orthologue in *S. praecaptivus* (Sant_) given for reference. The genes encoding the d-glucose-specific PtsG and MalX are likely pseudogenized. *S. glossinidius* has retained the ability to transport GlcNAc and mannitol through the specific NagE and MtlA systems, respectively. Other carbon sources can be imported via the promiscuous ManXYZ.

d-Glucose and other saccharides were investigated for their potential to act as the main carbon source for *S. glossinidius*. The reannotated *S. glossinidius* genome showed that the glucose-specific phosphotransferase system (PTS) gene *ptsG* had been pseudogenized. It is intact in *S. praecaptivus* (*Sant_2470*), suggesting that there may be weak selection for its retention within the tsetse. While the nonspecific ManXYZ could substitute for this loss of function (Fig. 3), alternative carbon sources were examined computationally and experimentally.

This investigation highlighted immediately the presence of a GlcNAc-specific PTS transporter gene, *nagE*, in *S. glossinidius* (*SG0859*) (18) (Fig. 3). The maintenance of *nagE* alongside the promiscuous ManXYZ implies that GlcNAc could be an important carbon source. GlcNAc is also of particular interest with regard to tsetse biology. It is a breakdown product of the insect’s chitinous peritrophic membrane and a potential link with the persistence of trypanosome infection (15, 37, 38). The mannitol-specific transporter encoded by *mtlA* (*SG0014*) is also retained (18) (Fig. 3). When used as the main carbon source, *i*LF517 produced biomass output values of 0.30, 0.35, and 0.32 g DW (mmol glucose)^−1^ h^−1^ for d-glucose, GlcNAc, and mannitol, respectively.

*S. glossinidius* was grown experimentally in LB and LB supplemented with a selection of carbon sources to test the hypothesis that GlcNAc and mannitol may be suitable alternatives. Of the saccharides tested, only d-glucose, GlcNAc, and mannitol increase growth significantly in comparison to LB alone (Fig. 2B) (*P* < 0.01 by one-way ANOVA). Approximately two times greater endpoint growth is exhibited with an equimolar amount of GlcNAc compared to d-glucose (*P* < 0.05 by one-way ANOVA) (Fig. 2B). Normalizing the carbon added from d-glucose with regard to GlcNAc has no significant effect on the optical density reached (data not shown). This demonstrates that the difference in growth between d-glucose and GlcNAc is not a result of the additional carbon. *S. glossinidius* grows significantly better on GlcNAc than mannitol (*P* < 0.05 by one-way ANOVA), reflecting the *in silico* results qualitatively.

### *S. glossinidius* has adapted to thiamine produced by the primary tsetse symbiont.

The addition of l-arginine, l-glutamate, and a carbon source, namely, GlcNAc, does not produce a positive biomass output in *i*LF517. It is likely that *S. glossinidius* requires supplementation from certain vitamins that it cannot synthesize. During the transition to symbiosis, *S. glossinidius* may lose genes that encode components of the vitamin and cofactor biosynthetic pathways in favor of retaining transporters. There is also thought to be a connection between aspects of the tsetse microbiome in terms of vitamin biosynthesis (19, 39).

*S. glossinidius* appears to have retained the components of pantothenate, biotin, riboflavin, protoheme, NAD, PLP, and tetrahydrofolate biosynthesis pathways found in *S. praecaptivus* (Table 1 and Table S1). Neither *S. praecaptivus* nor *S. glossinidius* can synthesize cobalamin. The key difference between the two species is in the pathway for thiamine biosynthesis. The loss of this pathway in *S. glossinidius* has been noted previously (39), but here the complete pathway in *S. praecaptivus* (*Sant_3916-21*) is used as a comparison. The loss of this pathway in *S. glossinidius* may be a specific adaptation to the tsetse. To investigate this further, three other *Sodalis*-allied symbionts were examined: “*Candidatus* Sodalis pierantonius” strain SOPE from the rice weevil Sitophilus oryzai (40, 41), and *Sodalis*-like symbionts from the meadow spittlebug Philaenus spumarius (42) and the seed bug Henestaris halophilus (43). The P. spumarius symbiont appears most similar to *S. praecaptivus*, with the fewest number of genes predicted to be absent or pseudogenized, whereas the H. halophilus symbiont has lost the ability to encode the components of the entire biotin and protoheme biosynthetic pathways (Table 1). “*Ca.* Sodalis pierantonius” differs from *S. glossinidius* in that it cannot synthesize biotin (Table 1). However, this organism, along with the *H. halophilus* symbiont, does share similarities with *S. glossinidius* in the pseudogenization of genes encoding components of the thiamine pathway, including *thiF*, *thiG*, and *thiH*. This suggests an adaptation to symbiosis with certain insects. It is important to note that *S. glossinidius* is unusual in that it can function as either a primary or secondary symbiont depending on the insect host, and therefore, interspecies comparisons should be treated with caution (44). Thiamine is a cofactor for many enzymes, including pyruvate dehydrogenase (45), that are essential in *i*LF517. The potential thiamine auxotrophy in *S. glossinidius* was assessed *in silico*, and supplementation of thiamine or TMP was required to produce a positive biomass output in *i*LF517 (Data Set S1).

**TABLE 1:**
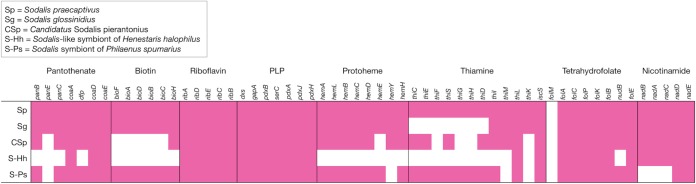
Completeness of vitamin biosynthesis pathways in *Sodalis* species^*a*^

a The presence (pink) or absence (white) of vitamin biosynthesis genes, found using tBLASTn and BLASTp, in *S. praecaptivus* (Sp), *S. glossinidius* (Sg), “*Candidatus* Sodalis pierantonius” (Csp), *Sodalis*-like symbiont of *Henestaris halophilus* (S-Hh), and *Sodalis* endosymbiont of *Philaenus spumarius* (S-Pf).

10.1128/mBio.02106-18.5 TABLE S1 Vitamin biosynthesis capabilities. BLASTp results showing orthologues of *S. praecaptivus* vitamin biosynthesis proteins in *S. glossinidius*. Download Table S1, DOCX file, 0.01 MB. Copyright © 2019 Hall et al. 2019 Hall et al. This content is distributed under the terms of the Creative Commons Attribution 4.0 International license.

The reliance of *S. glossinidius* on an external source of thiamine in order to produce TMP was then investigated experimentally. TMP was removed from SGM11, and the ability of the symbiont to grow was measured. Removal of TMP from SGM11 resulted in a significant reduction in *S. glossinidius* growth (*P* < 0.05 by one-way ANOVA) (Fig. 2C). A thiamine ABC transporter (SG0431-3) that likely transports TMP is also present (46). This adds weight to the *in silico* evidence from *i*LF517 and the elegant empirical work of Snyder et al. (86) that *S. glossinidius* relies on TMP from its environment. The only source of TMP in the tsetse microenvironment is that excreted by *W. glossinidia*, which is thought to supply it to the tsetse. *S. glossinidius* may therefore have adapted to not only its host but also to the metabolism of the primary symbiont in order to scavenge the available TMP.

### *S. glossinidius* is dependent on external sources of l-glutamate or l-aspartate.

Culturing *S. glossinidius* in SGM11 enables the thorough testing of amino acid usage in *i*LF517. Metabolites can be removed individually, and the effect on biomass production can be measured. *i*LF517 requires either l-glutamate or l-aspartate to produce a positive biomass output. Both l-glutamate and l-aspartate likely enter the cell through the GltP transporter SG2121 (Fig. 1). *S. glossinidius* is predicted to have transporters for 14 amino acids (Table S2), so this reliance on l-glutamate or l-aspartate is not merely due to the ability to transport only these amino acids.

10.1128/mBio.02106-18.6 TABLE S2 Amino acid biosynthesis and transport capabilities. The transporter families (TransportDB) are dicarboxylate/amino acid:cation symporter (DAACS), amino acid-polyamine-organocation family (APC), branched-chain amino acid symporter (LIVSC), alanine or glycine:cation symporter (AGCS), and hydroxyl/aromatic amino acid permease (HAAAP). Download Table S2, DOCX file, 0.01 MB. Copyright © 2019 Hall et al. 2019 Hall et al. This content is distributed under the terms of the Creative Commons Attribution 4.0 International license.

Removing l-aspartate or l-glutamate from SGM11 individually resulted in a significant decrease in the growth yield achieved by *S. glossinidius* after incubating for 72 h (*P* < 0.05 by one-way ANOVA) (Fig. 2D). Removing both l-aspartate and l-glutamate abolished growth completely (*P* < 0.001 by one-way ANOVA). This confirms that an exogenous source of one of these amino acids is essential for *S. glossinidius* growth. Examination of *i*LF517 reveals that l-glutamate feeds directly into the TCA cycle through deamination to α-ketoglutarate. The direct route to feed l-aspartate into the TCA cycle via fumarate (l-aspartase) is however missing in *S. glossinidius* (Fig. 1). When *i*LF517 is supplied with l-aspartate instead of l-glutamate, 69% of the available l-aspartate is channelled into the aspartate transaminase reaction (ASPTA), producing l-glutamate and oxaloacetate. The resulting biomass output was reduced from 0.35 to 0.31 g DW (mmol glucose)^−1^ h^−1^, demonstrating that *S. glossinidius* can use l-aspartate if l-glutamate is not available, although the latter may be preferred. l-Glutamate is therefore likely an important energy source in *i*LF517 both to form l-aspartate and to replenish the TCA cycle at α-ketoglutarate (Fig. 1).

### *S. glossinidius* is not an l-arginine auxotroph.

Initial analysis of amino acid biosynthesis in *S. glossinidius* appeared to confirm existing opinion (29) that the only amino acid with an incomplete biosynthetic pathway is l-arginine (Table S2). A functional uptake system is also present, suggesting that *S. glossinidius* is indeed an l-arginine auxotroph. To assess this experimentally, *S. glossinidius* was grown in SGM11 with l-arginine removed. The growth yield decreased (Fig. 2E), but surprisingly, it was not totally abolished as expected for a true auxotroph. Excess l-glutamate was added to SGM11 to determine whether it could rescue this reduction in growth, as l-glutamate appears to be a key metabolite to *S. glossinidius*. A fivefold increase in l-glutamate concentration to 85 mM increased the bacterial yield (Fig. 2E), likely due to the extra carbon and nitrogen available. Remarkably, the excess l-glutamate rescued the growth defect caused by the removal of l-arginine completely (Fig. 2E and Fig. S4).

10.1128/mBio.02106-18.4 FIG S4 Effects of l-arginine biosynthesis gene deletions on E. coli growth *in vitro*. Wild-type E. coli strain BW25113 (blue) was grown in M9 medium alone (solid line) or with added l-arginine (dashed line), l-glutamate (solid line, circle marker) or 5× concentration l-glutamate (dashed line, triangle marker) for 24 hours in a microplate reader. This was compared to growth in the same medium combinations for *argA* (pink), *argD* (orange), and *argG* (green) gene deletion mutants from the Keio collection (51). Two isolates for each mutant are shown. The *argD* knockout strain is able to grow under all conditions, whereas strains lacking *argA* and *argG* can grow only when exogenous l-arginine is provided. Measurements for triplicate experiments are shown; error bars depict SEM. The graph was produced using Plate Whisperer software for microplate data analysis, developed by Stephen Thorpe. Download FIG S4, TIF file, 3.2 MB. Copyright © 2019 Hall et al. 2019 Hall et al. This content is distributed under the terms of the Creative Commons Attribution 4.0 International license.

The *S. glossinidius* pathway for l-arginine biosynthesis was subsequently reanalyzed using the latest genome annotation (GenBank accession no. LN854557) to assess its completeness in comparison to *S. praecaptivus*. This revealed that *argB*, *argE*, *argF/argI*, and *argH* are full length and therefore likely functional (Table 2). It has been noted previously that *argC* is pseudogenized (29), but the new annotation indicates that this may not be the case. ArgC is also detected by proteomics, suggesting that this gene is indeed likely functional (47).

**TABLE 2:** Functionality of the l-arginine biosynthesis pathway^*a*^

Gene	Size^*b*^ in *E. coli*	Size in *S. praecaptivus*	Reaction	*S. glossinidius*^*c*^	Conclusion
*argA*	1,332 bp	Sant_0864	ACGS	***SGGMMB4_04654 (argA_1)***	*argA_2* may produce a subunit that can function individually, using its GTG start codon
	443 aa	418 aa		***SGGMMB4_04655 (argA_2)***	
				***804 bp total***	

*argB*	777 bp	Sant_3956	ACGK	*SGGMMB4_05193*	New annotation indicates full-length gene
	258 aa	257 aa		*765 bp*	

*argC*	1,005 bp	Sant_3957	AGPR	***SGGMMB4_05194***	New annotation indicates full-length gene and ArgC detected by proteomics
	334 aa	334 aa		***999 bp***	

*gabT*	1,281 bp	Sant_2160	ACOTA	***See argD***	See *argD*
	426 aa	425 aa			

*argD*	1,221 bp	Sant_0398	ACOTA	***SGGMMB4_05438 (argD_1)*** ***1–708 bp***	May use functional alternatives *bioA* (*SG0902*) or *hemL* (*SG0500*)
	406 aa	407 aa		***SGGMMB4_05439 (argD_2)*** *** 681–828 bp***	

*argE*	1,152 bp	Sant_3958	ACODA	*SGGMMB4_05195*	New annotation indicates full-length gene
	383 aa	382 aa		*1,146 bp*	

*argF*	1,005 bp	Sant_3829	OCBT	***SGGMMB4_05057***	New annotation indicates full-length gene
	334 aa	338 aa		***1,014 bp***	

*argI*	1,005 bp	Sant_3829	OCBT	***SGGMMB4_05057***	New annotation indicates full-length gene
	334 aa	338 aa		***1,014 bp***	

*argG*	1,344 bp	Sant_2433	ARGSS	***SGGMMB4_03589 (argG_1)***	New annotation indicates *argG_2* fragment is almost full length
	447 aa	445 aa		***SGGMMB4_03590 (argG_2)***	
				***1,341 bp total***	

*argH*	1,374 bp	Sant_3955	ARGSL	*SGGMMB4_05192*	New annotation indicates full-length gene
	457 aa	457 aa		*1,371 bp*	

a tBLASTn results for *S. glossinidius* orthologues of components of the l-arginine biosynthesis pathway in E. coli and *S. praecaptivus*.

b aa, amino acids.

c Functional orthologues are indicated by italic boldface type, and those components for which the new *S. glossinidius* genome annotation has provided evidence for functionality are shown in italic type.

The new annotation suggests that the *S. glossinidius argG* gene (SGGMMB4_03590) has a fragment (*argG_2*) that is almost full length in comparison to its *S. praecaptivus* orthologue. This indicates that *argG* is also likely functional in spite of its description as a pseudogene in the previous annotation.

The *argA* gene appears in two fragments: SGGMMB4_04654 (*argA_1*) and SGGMMB4_04655 (*argA_2*). It has been demonstrated in Pseudomonas aeruginosa that the two separate ArgA protein domains can be expressed individually (48, 49). The C-terminal acetyltransferase domain can also function as a stand-alone protein when a high concentration of l-glutamate is provided (48). The SGGMMB4_04655 (*argA_2*) fragment of this gene has a GTG start codon and may therefore be functional under the conditions shown in Fig. 2E.

**FIG 4:**
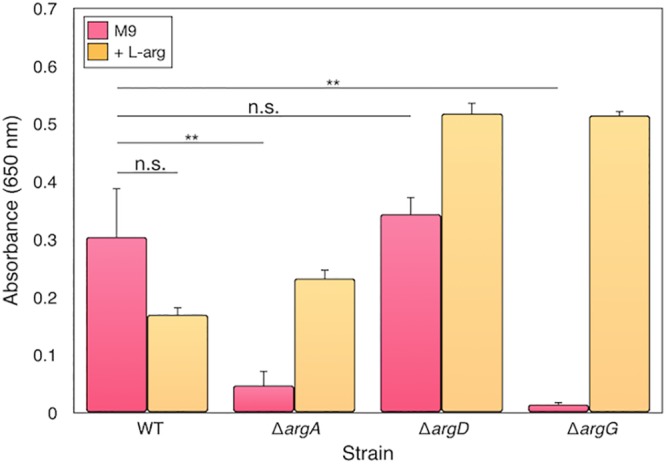
Using E. coli knockout mutants from the Keio collection to examine the essentiality of l-arginine biosynthesis genes. E. coli *argA*, *argD*, and *argD* deletion mutants grown in M9 plus glucose (pink) with the addition of l-arginine (orange), in comparison to wild-type (WT) strain BW25113. *argA* and *argG* knockout mutants are not able to grow in the absence of exogenous l-arginine, whereas the WT and the *argD* knockout mutant can grow. The measurements are endpoint growth increase in triplicate, and the error bars show SEM. For time course results, see Fig. S4 in the supplemental material.

The *S. glossinidius argD* orthologue also appears in two pieces: SGGMMB4_05438 (*argD_1*) and SGGMMB4_05439 (*argD_2*). Lal et al. (50) showed that an *argD* mutant of E. coli can still exhibit some *N*-acetylornithine aminotransferase activity, demonstrating that other proteins can compensate for a loss of this gene. The hypothesis that the loss of certain genes in the l-arginine biosynthesis pathway is not lethal was then assessed *in vivo*. E. coli *argA*, *argD*, and *argG* knockouts from the Keio collection (51) were grown in M9 minimal medium (36) alone or with the addition of l-arginine. The *argD* knockout mutant can grow in the absence of l-arginine (Fig. 4), confirming that the loss of this gene can be compensated for by alternative proteins in E. coli. Indeed, candidate aminotransferase genes exist in *S. glossinidius*, including *bioA* (*SG0902*) or *hemL* (*SG0500*), that may provide functional alternatives.

The data here suggest that under certain conditions, *S. glossinidius* can synthesize l-arginine, surviving when it is not supplied exogenously. Therefore, it is not a true auxotroph and could instead be described as a relic of a prototroph transitioning to auxotrophy.

## DISCUSSION

Symbiotic bacteria are important components of medically significant microbiomes. However, studying their physiology and metabolism is limited frequently by culturability issues. Tools to improve this are therefore desirable. This work describes a new refined FBA model of the *S. glossinidius* metabolic network and demonstrates its application in designing defined growth media for the symbiont. The carbon source for *i*LF517 is GlcNAc, as opposed to d-glucose in *i*EB458. It has been verified empirically that *S. glossinidius* achieves a significantly better growth yield with GlcNAc than with d-glucose (Fig. 2B). This is an important progression in the development of a metabolic model of *S. glossinidius*. The use of GlcNAc may be a result of both the pseudogenization of the glucose-specific PTS transporter (Fig. 3) and the availability *in vivo* of this host-derived sugar. This inclusion of GlcNAc may also support the theory that *S. glossinidius* is connected to the persistence of the trypanosomes within the tsetse. GlcNAc can inhibit d-glucose uptake by procyclic trypanosomes, resulting in a metabolic switch to the more-efficient oxidative phosphorylation with l-proline and a higher growth rate (38, 52). The free GlcNAc may derive from a breakdown of the tsetse peritrophic membrane by a chitinase secreted by *S. glossinidius* (15). The data here indicate that there may indeed be a link between the symbiont, the parasite, and the availability of GlcNAc within the tsetse.

The experimental evidence demonstrates that *S. glossinidius* still requires a sugar for growth, even though it clearly relies on amino acids such as l-glutamate (Fig. 2D). This is important, as other bacterial species have been shown to reduce their metabolic networks to grow on amino acids alone (53–57). The fact that this is not the case suggests an adaptation to use an abundant host-derived sugar, namely, GlcNAc. Furthermore, this may have then allowed the loss of *ppc* to occur during the transition to a symbiotic lifestyle (Fig. 1). The result is a more-constrained metabolic network that makes the organism less metabolically flexible than its free-living relative.

l-Glutamate has been shown *in silico* and empirically to be an essential nutrient for *S. glossinidius* (Fig. 2D; see also Data Set S1 in the supplemental material). It supplements the TCA cycle at α-ketoglutarate and forms l-arginine via ornithine (Fig. 1). An excess of this amino acid rescued the growth defect caused by the removal of l-arginine, previously thought to be an essential metabolite (Fig. 2E). The *argD* gene has become fragmented, but all other genes thought previously to be pseudogenized appear functional in the new genome annotation (Table 2). *S. glossinidius* is therefore an l-arginine prototroph, not an auxotroph as thought previously, capable of growth in the absence of l-arginine when sufficient l-glutamate is available. Unusual amino acid biosynthesis pathways are not uncommon in symbiotic bacteria. Indeed, components of the l-arginine biosynthesis pathway can function differently in symbionts. One example is a potential fusion of ArgA and ArgG in Sulcia muelleri, symbiont of the sap-feeding sharpshooter Homalodisca vitripennis (58). The experimental conditions used here aim to reflect the tsetse microenvironment; metabolite concentrations vary according the stage of the hunger cycle or the tissue sampled, but internal l-glutamate has been measured at 34 mM in the tsetse tissue (59, 60). SGM11 could be considered “low” l-glutamate at 18 mM, and therefore, the 5× (85 mM) medium subsequently removes any limitations caused by insufficient L-glutamate.

It may be that the l-arginine biosynthesis pathway is undergoing the process of inactivation and will become entirely pseudogenized over evolutionary time. This is supported by the complete pathway for l-arginine biosynthesis in *S. praecaptivus*, suggesting that these genes may have been lost within the tsetse environment as a result of selection pressure or drift. It implies that l-glutamate is not limiting inside the tsetse, allowing relaxed selection pressure on the l-arginine biosynthesis genes. It also emphasizes strongly the importance of using *in vitro* experiments to test *in silico* assertions. This is particularly relevant in symbiotic bacteria where the functionality of broken or fragmented genes is not certain. Indeed, a recent report implies that some *S. glossinidius* genes thought to be pseudogenized are in fact under transcriptional control (47).

TMP has been described here as an essential external metabolite. *S. glossinidius* is reliant on an external source of thiamine, both *in silico* in *i*LF517 and experimentally in the form of TMP in SGM11 (Fig. 2C). *S. glossinidius* may use its intact transporter to obtain TMP *in vivo* from *W. glossinidia*, which has retained the ability to synthesize this vitamin (18, 39). The results presented here provide the first clear experimental evidence of a potential metabolic linkage between the two important symbionts of the tsetse, *S. glossinidius* and *W. glossinidia*, and suggest that the TMP released by *W. glossinidia* is transported around the tsetse for use by both host cells and other symbionts.

*i*LF517 and SGM11 can now be used as a tool to predict with accuracy how *S. glossinidius* might respond to genetic manipulation. Using genomics to investigate and implement custom growth conditions is an area of research that is progressing rapidly, aided by advancements in gene sequencing and analysis. This includes the design of defined microbiological growth media (61–63), enabling metabolic and physiological investigations that would not be possible with complex or standard media (64–67). This has implications in disease control, both for HAT and for other diseases where the insect vectors have characterized bacterial microbiomes (68, 69). *Wolbachia*, for example, has been introduced successfully into the mosquito Aedes aegypti, with a notable reduction in infection by the pathogenic dengue and chikungunya viruses and the malaria parasite *Plasmodium* (70, 71). *Wolbachia* is found naturally in a range of medically significant insects, including Phlebotomus chinensis (visceral leishmaniasis) (72) and Aedes albopictus (dengue, yellow fever, West Nile, and chikungunya) (73–75). While some tsetse populations do present with *Wolbachia* infection (76–79), the persistence of *S. glossinidius* and its colocalization with the parasitic T. brucei make the latter an ideal candidate for novel disease control methods. It is hoped that the techniques described here may also translate to the microbiomes of other medically significant insects, including Rhodococcus rhodnii from the Chagas disease vector Rhodnius prolixus (80), and *Acetobacteraceae* spp. and *Pseudomonadaceae* spp. in Leishmania infantum-infected sand flies (Lutzomyia longipalpis) (81).

Use of *i*LF517 and the *S. glossinidius* metabolic network has enabled the design of a defined growth medium that supports growth of the symbiont. While several FBA models for insect symbionts have been published (82–85), this study is the first example of using FBA to improve the *in vitro* culture of these organisms. SGM11 facilitated the discovery that *S. glossinidius* is not a true l-arginine auxotroph and demonstrates its reliance on exogenous sources of thiamine and l-glutamate. SGM11 will greatly improve the ability to test other aspects of *S. glossinidius* metabolism and growth kinetics that have until now been limited by the restrictions of rich media. The continued transition of *S. glossinidius* to a symbiotic lifestyle can now be predicted using this model. By comparing its dispensable, redundant genes to those in both free-living and symbiotic bacteria, it is possible to assess the trajectory of this symbiosis.

## MATERIALS AND METHODS

### Refinement of the *S. glossinidius* metabolic network.

The previously published whole-genome metabolic model (GEM) *i*EB458 (29) was assessed for missing or potentially incorrect gene assignments. A reaction was removed if there could be no functional gene identified, either through absence or through pseudogenization based on the size of the gene in comparison to its E. coli or *S. praecaptivus* orthologues. Those reactions for which a gene assignment had been uncovered were added to the new model. Reactions were maintained if removing them resulted in a lethal phenotype, observed when the biomass output returned a value of zero. BiGG Models, KEGG, and EcoCyc databases were used to identify E. coli genes encoding the reactions for which an *S. glossinidius* gene assignment had not been found. Translated nucleotide and protein BLAST searches were used to look for known *S. glossinidius* proteins, and confirmation of genes and pseudogenes was performed using the Artemis genome visualization tool. Candidate pseudogenes were aligned with functional orthologues using ClustalX2.

### Flux balance analysis.

The flux balance analysis (FBA) solutions were generated using the GNA linear programming kit (GLPK) integrated with custom software in Java. Oxygen uptake was constrained to 12 mmol g DW^−1^ h^−1^ in order to simulate a reduced oxygen environment. The uptake of ammonia, water, phosphate, sulfate, potassium, sodium, calcium, carbon dioxide, protons, and essential transition metals was unconstrained. Uptake of all other metabolites was set at zero, with the exception of those used in the analyses which have been set at 2 mmol g DW^−1^ h^−1^ GlcNAc and l-glutamate, 0.5 mmol g DW^−1^ h^−1^ l-arginine, and 0.01 mmol g DW^−1^ h^−1^ thiamine. Cofactor constraints were implemented by introducing these metabolites to the biomass functions at small fluxes (0.00001 mmol g DW^−1^ h^−1^) (82). The phenotype was considered viable if the biomass production rate was greater than 1 × 10^−1^ g DW (mmol glucose)^−1^ h^−1^. Futile cycles, identified as reactions carrying biochemically unsustainable flux, were altered to the correct reaction stoichiometry where possible with guidance from EcoCyc and BiGG. The full description of the model is provided in Data Set S1 in the supplemental material.

### Bacterial strains, growth conditions, and reagents.

*S. glossinidius* strain GMM4 was obtained from the University of Liverpool. Working stocks were established by growing starter cultures on brain heart infusion (BHI) (Sigma-Aldrich) plates under microaerophilic conditions generated by Oxoid CampyGen sachets (Thermo Fisher Scientific) until growth was visible. Colonies were then transferred to liquid BHI medium and incubated for 4 to 7 days in cell culture flasks at room temperature until growth was visible. *S. glossinidius* from the working stock was then transferred to 5 ml fresh Luria-Bertani (LB) medium (Sigma-Aldrich) and supplemented with either d-glucose or GlcNAc (Sigma-Aldrich).

*i*LF517 and the *S. glossinidius* metabolic network were used to design *in silico* an entirely defined medium, SGM11, in which to grow the bacterium. *S. glossinidius* from the working stock was transferred to 5 ml of M9 minimal medium (36) containing the following supplements; 17 mM GlcNAc, 17 mM trehalose, 17 mM l-serine, 17 mM l-arginine, 4 mM l-proline, 17 mM l-glutamic acid monosodium salt hydrate, 17 mM l-aspartate, 4 mM nicotinamide, 9 mM α-ketoglutarate, 9 mM fumaric acid, and 0.4 mM thiamine monophosphate (Sigma-Aldrich). Metabolites were omitted from SGM11 individually and in combination to test the model predictions.

The culture flasks were incubated for 48 h (LB) or 72 h (SGM11) at 25°C in a temperature-controlled water bath. Gentle agitation was achieved using magnetic stir bars to achieve a suitable balance between oxygenation, settling, and disturbance, and a stirring speed of 500 rpm was used. Intermediate time points were found to compromise the sterility of the cultures, and therefore, destructive sampling was the only reliable method of investigation, negating the possibility of higher-resolution temporal data. Endpoint increase in *S. glossinidius* growth was measured at an optical density of 650 nm. Preliminary experiments using variable sampling times indicated that this was the most appropriate sampling time for *S. glossinidius* to reproducibly capture the final steady state but still retain a proxy for growth rate to guide the modeling results.

E. coli gene deletion mutants were obtained from the Keio collection (51). Cells were cultured in M9 minimal medium (34) with 0.4% d-glucose, and either 20 or 100 mM l-glutamic acid monosodium salt hydrate or 20 mM l-arginine in a microplate reader for 24 h at 37°C.

### Flow cytometry.

Cell count was generated using *S. glossinidius* taken from a starter culture in BHI and diluted in M9 salts. Cells were stained with DAPI at 2 μl/ml for 10 min at room temperature and measured on the CytoFLEX S flow cytometer (Fig. S3). The flow cytometer was calibrated using counting beads from Beckman Coulter (Miami, FL, USA).

### Statistical analysis.

All statistical analyses were performed using SciPy in Python (version 2.7.10, www.python.org). Error bars show standard errors of the means, and statistical significance was assessed using one-way analysis of variance (ANOVA).
