# Supplementary figures and images for: A Tale of Three Species: Adaptation of Sodalis glossinidius to Tsetse Biology, Wigglesworthia Metabolism, and Host Diet
Source: mBio. 2019 Jan 2;10(1):e02106-18. doi: 10.1128/mBio.02106-18 (PMC6315101; doi:10.1128/mBio.02106-18)

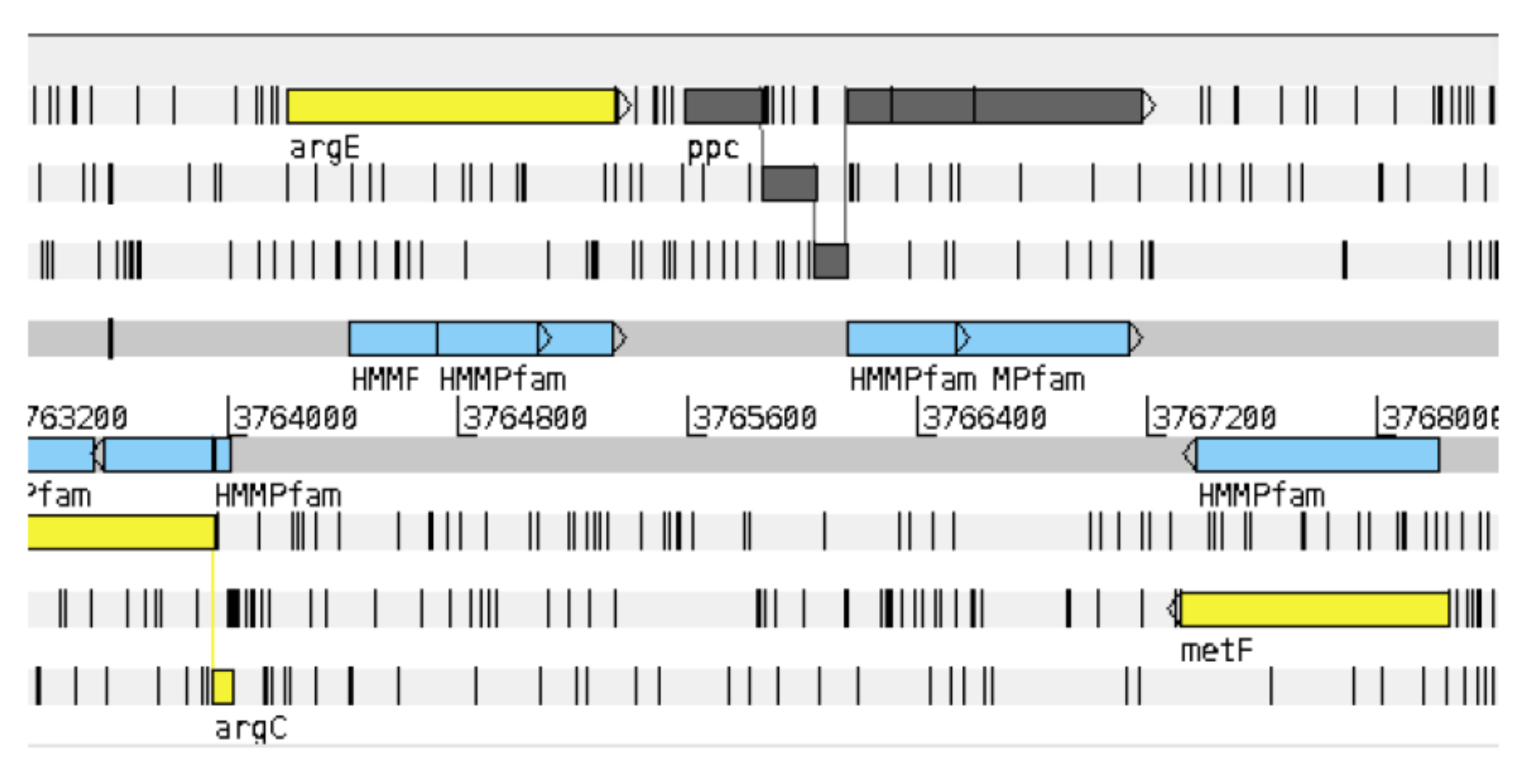

Supplement: FIG S1 [file mbo006184240sf1.tif]

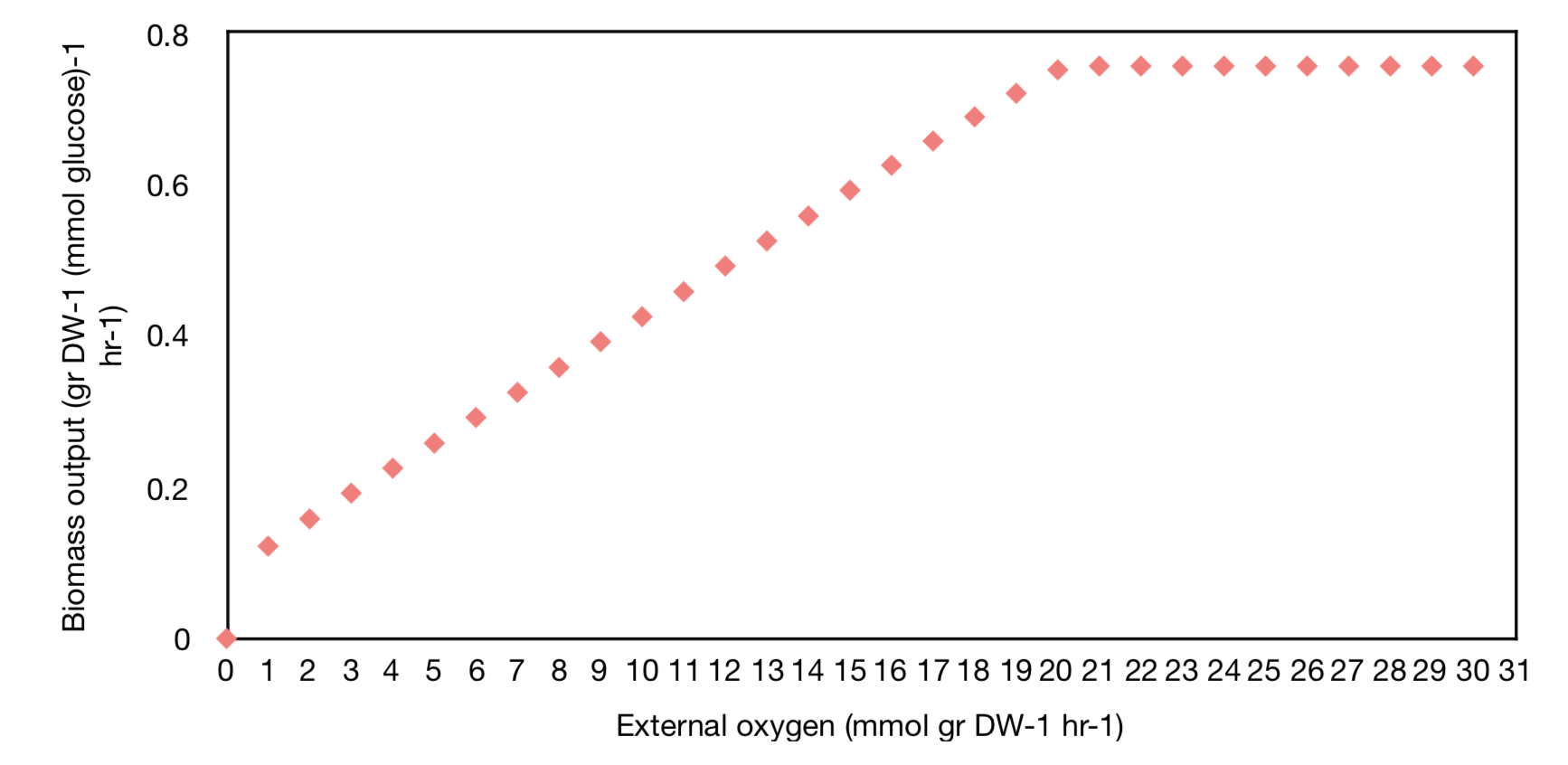

Supplement: FIG S2 [file mbo006184240sf2.tif]

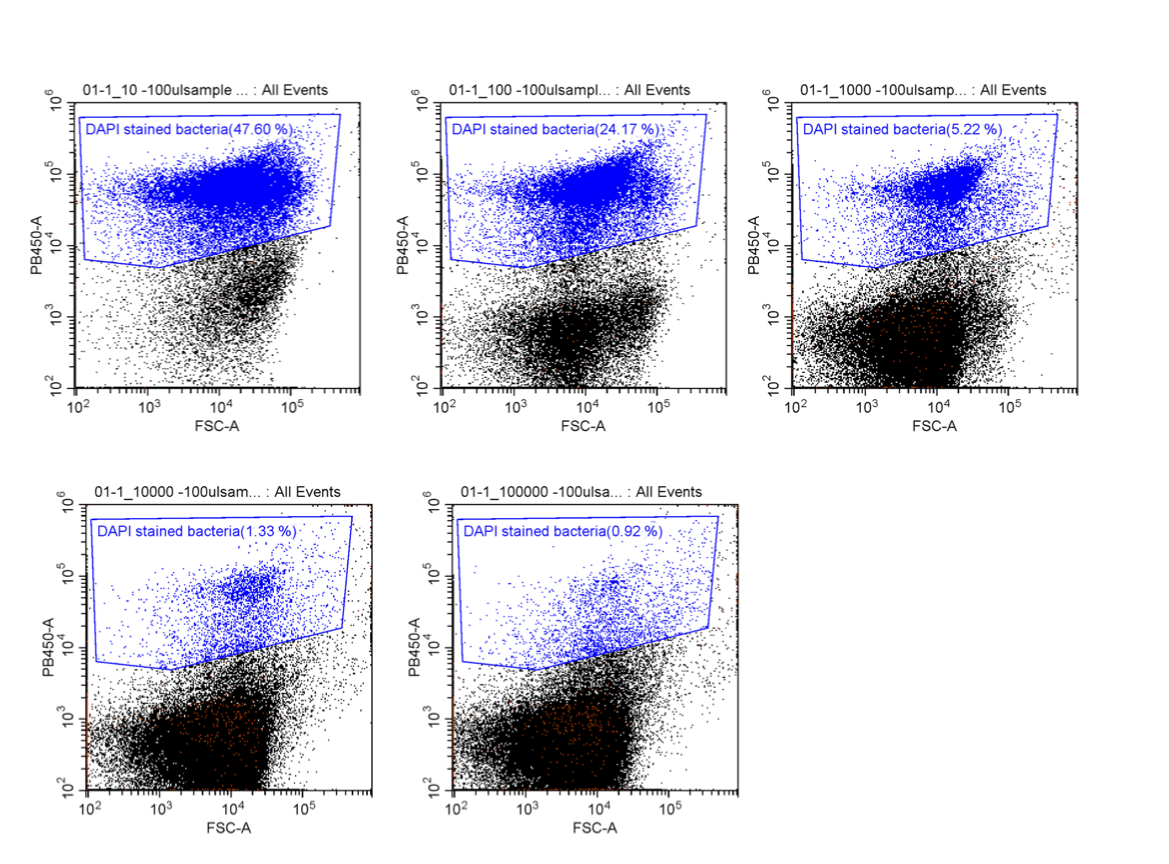

Supplement: FIG S3 [file mbo006184240sf3.tif]

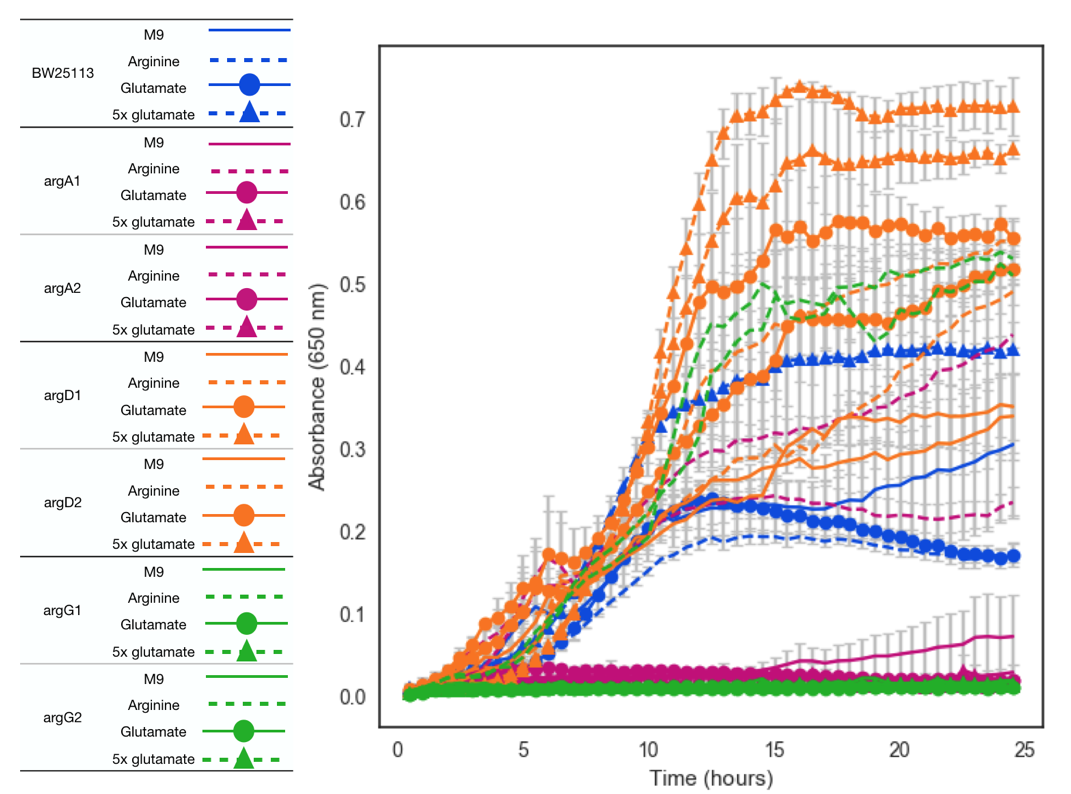

Supplement: FIG S4 [file mbo006184240sf4.tif]
