# Supplementary material for: A Tale of Three Species: Adaptation of Sodalis glossinidius to Tsetse Biology, Wigglesworthia Metabolism, and Host Diet
Source: mBio. 2019 Jan 2;10(1):e02106-18. doi: 10.1128/mBio.02106-18 (PMC6315101; doi:10.1128/mBio.02106-18)
Supplement: TABLE S1 [file mbo006184240st1.docx]

| **Table S1**  Vitamin biosynthesis capabilities. BLASTp results showing orthologues of *S. praecaptivus* vitamin biosynthesis proteins in *S. glossinidius*. | | | | | |
| --- | --- | --- | --- | --- | --- |
| **Vitamin** | **Gene** | **Reaction** | **BiGG reaction** | ***S. glossinidius*** | ***S. praecaptivus*** |
| Pantothenate | *panB* | 3-methyl-2-oxobutanoate hydroxymethyltransferase | MOHMT | SG0488 | Sant_3309 |
|  | *panE* | 2-dehydropantoate 2-reductase | DPR | SG0661 | Sant_3012 |
|  | *panC* | Pantothenate synthase | PANTS | SG0487 | Sant_3310 |
|  | *coaA* | Pantothenate kinase | PNTK | SG0126 | Sant_3935 |
|  | *dfp* | Phosphopantothenate-cysteine ligase | PPNCL2 | SG2209 | Sant_4020 |
|  | *dfp* | Phosphopantothenoylcysteine decarboxylase | PPCDC | SG2209 | Sant_4020 |
|  | *coaD* | Pantetheine-phosphate adenylyltransferase | PTPATi | SG2205 | Sant_4015 |
|  | *coaE* | Dephospho-CoA kinase | DPCOAK | SG0461 | Sant_3340 |
|  | *panF* | Pantothenate sodium symporter | PNTOt4pp | — | Sant_0493 |
| Biotin | *bioF* | 8-amino-7-oxononanoate synthase | AOXSr | SG0904 | Sant_2734 |
|  | *bioA* | Adenosylmethionine-8-amino-7- oxononanoate transaminase | AMAOTr | SG0902 | Sant_2736 |
|  | *bioD* | Dethiobiotin synthase | DBTS | SG1466 | Sant_2732 |
|  | *bioB* | Biotin synthase | BTS2 | SG0903 | Sant_2735 |
|  | *bioC* | Malonyl-CoA methyltransferase | MALCOAMT | SG0905 | Sant_2733 |
|  | *bioH* | Pimeloyl-[ACP] methyl ester esterase | PMEACPE | SG2324 | Sant_0363 |
| Riboflavin | *ribA* | GTP cyclohydrolase II | GTPCII | SG1410 | Sant_1871 |
|  | *ribD* | 5-amino-6-(5-  phosphoribosylamino)uracil reductase | APRAUR | SG0651 | Sant_3023 |
|  | *ribE* | Riboflavin synthase | RBFSb | SG1439 | Sant_3022 |
|  | *ribC* | Riboflavin synthase | RBFSa | SG1439 | Sant_2045 |
|  | *ribB* | 3,4-dihydroxy-2-butanone-4- phosphate synthase | DB4PS | SG0263 | Sant_3614 |
| Protoheme | *hemA* | Glutamyl-tRNA reductase | GLUTRR | SG1877 | Sant_2113 |
|  | *hemL* | Glutamate-1-semialdehyde  aminotransferase | G1SAT | SG0500 | Sant_3285 |
|  | *hemB* | Porphobilinogen synthase | PPBNGS | SG1529 | Sant_2301 |
|  | *hemC* | Hydroxymethylbilane synthase | HMBS | SG2366 | Sant_0327 |
|  | *hemD* | Uroporphyrinogen-III synthase | UPP3S | SG2367 | Sant_0326 |
|  | *hemE* | Uroporphyrinogen decarboxylase | UPPDC1 | SG0138 | Sant_3913 |
|  | *hemY* | Protoporphyrinogen oxidase | PPPGO | SG0121 | Sant_0261 |
|  | *hemH* | Ferrochelatase | FCLT | SG0694 | Sant_2970 |
|  | *ccmABCDE* | Protoheme transport via ABC system | PHEMEabcpp | SG1635-7 (*ccmBE* orthologues pseudogenised) | Sant_1288-92 |
| PLP | *dxs* | 1-deoxy-D-xylulose 5-phosphate synthase | DXPS | SG0656 | Sant_3017 |
|  | *gapA* | Erythrose 4-phosphate dehydrogenase | E4PD | SG1347 | Sant_1815 |
|  | *pdxB* | Erythronate 4-phosphate dehydrogenase | PERD | SG1621 | Sant_1314 |
|  | *serC* | O-phospho-4-hydroxy-L-threonine: 2-oxoglutarate aminotransferase | OHPBAT | SG0990 | Sant_2634 |
|  | *pdxA, pdxJ* | Pyridoxine 5’-phosphate synthase | PDX5PS | SG0425, SG1784 | Sant_3384, Sant_1101 |
|  | *pdxH* | Pyridoxine 5’-phosphate oxidase, Pyridoxamine 5’-phosphate oxidase | PDX5POi, PYAM5PO | SG1447 | Sant_2034 |
| Thiamine | *thiC* | 4-amino-2-methyl-5- phosphomethylpyrimidine synthetase | AMPMS2 | — | Sant_3916 |
|  | *thiE* | Thiamine-phosphate diphosphorylase | TMPPP | — | Sant_3917 |
|  | *thiF* | Sulfur carrier protein adenylyltransferase | THZPSN | — | Sant_3918 |
|  | *thiS* | Sulfur carrier protein | THZPSN | — | Sant_3919 |
|  | *thiG* | Thiazole synthase | THZPSN | — | Sant_3920 |
|  | *thiH* | 2-iminoacetate synthase | THZPSN | — | Sant_3921 |
|  | *thiD* | Hydroxymethylpyrimidine kinase, phosphomethylpyrimidine kinase | HMPK1, PMPK | — | Sant_1177 |
|  | *thiI* | tRNA sulfurtransferase | THZPSN | SG0659 | Sant_3014 |
|  | *thiM* | Hydroxyethylthiazole kinase | HETZK | SG1739 | Sant_1176 |
|  | *thiL* | Thiamine-phosphate kinase | TMPK | SG0654 | Sant_3020 |
|  | *thiK* | Thiamine kinase | TMK | SG1071 | Sant_2466 |
|  | *iscS* | Cysteine desulfurase | THZPSN, ICYSDS | SG1769 | Sant_1125 |
|  | *tbpAthiPQ* | Thiamine transport via ABC system | THMabcpp | SG0431-3 | Sant_3371-3 |
| Tetrahydrofolate | *folM* | Dihydromonapterin reductase | DHMPTR | — | — |
|  | *folA* | Dihydrofolate reductase | DHFR | SG0421 | Sant_3388 |
|  | *folC* | Dihydrofolate synthase | DHFS | SG1616 | Sant_1319 |
|  | *folP* | Dihydropteroate synthase | DHPS2 | SG0372 | Sant_3460 |
|  | *folK* | 6-hydroxymethyl-dihydropterin pyrophosphokinase | HPPK2 | SG0489 | Sant_3308 |
|  | *folB* | Dihydroneopterin aldolase, dihydroneopterin epimerase | DHNPA2r, DHNPTE | SG0256 | Sant_3622 |
|  | *nudB* | Dihydroneopterin triphosphate pyrophosphatase | DNTPPA | SG1258 | Sant_1683 |
|  | *folE* | GTP cyclohydrolase I | GTPCI | SG0957 | Sant_1427 |
| Cobalamin | *btuR* | Cobinamide adenyltransferase, cob(I)alamin adenosyltransferase | CBIAT, CBLAT | — | Sant_1862 |
|  | *cobT* | Nicotinate-nucleotide dimethylbenzimidazole phosphoribosyltransferase | NNDMBRT | — | — |
|  | *cobC* | Alpha-ribazole 5-phosphate phosphatase | RZ5PP | — | — |
|  | *cobU* | Adenosyl cobinamide phosphate guanyltransferase, adenosyl  cobinamide kinase | ACBIPGT, ADOCBIK | — | — |
|  | *cobS* | Adenosylcobalamin 5’-phosphate synthase | ADOCBLS | — | — |
| Nicotinamide | *nadB* | L-aspartate oxidase | ASPO6 | SG1794 | Sant_1091 |
|  | *nadA* | Quinolinate synthase | QULNS | SG0889 | Sant_2754 |
|  | *nadC* | Quinolinate phosphoribosyltransferase | NNDPR | SG0464 | Sant_3335 |
|  | *nadD* | Nicotinate-mononucleotide adenylyltransferase | NMNAT | SG0800 | Sant_3420 |
|  | *nadE* | NAD+ synthetase | NADS1 | SG1866 | Sant_2100 |
