# Supplementary material for: A Tale of Three Species: Adaptation of Sodalis glossinidius to Tsetse Biology, Wigglesworthia Metabolism, and Host Diet
Source: mBio. 2019 Jan 2;10(1):e02106-18. doi: 10.1128/mBio.02106-18 (PMC6315101; doi:10.1128/mBio.02106-18)
Supplement: TABLE S2 [file mbo006184240st2.docx]

| **Table S2** Amino acid biosynthesis and transport capabilities. Transporter families (TransportDB); dicarboxylate/amino acid:cation symporter (DAACS), amino acid-polyamine-organocation family (APC), branched chain amino acid symporter (LIVSC), alanine or glycine:cation symporter (AGCS), hydroxyl/aromatic amino acid permease (HAAAP). *Cystine is the oxidised form of cysteine and transported via the specific FliY, of which there is no apparent orthologue in *S. glossinidius* or *S. praecaptivus.* 1This is in contrast to Toh *et al*. (17) who state that the pathway of alanine biosynthesis is not complete. | | | | | |
| --- | --- | --- | --- | --- | --- |
| **Amino acid** | **Biosynthesis pathway completeness** | **Transport reaction** | **Transporter family** | **Gene assignment** | ***S. praecaptivus* transporter** |
| Glutamate | From α-ketoglutarate | GLUt2r | DAACS | *SG2121* | GLUt2r (Sant_3844)  GLUabc (Sant_2814-7) |
| Glutamine | From α-ketoglutarate | — | — | — | — |
| Arginine | **Incomplete** | ARGabc | ABC | *SG1093-6* | ARGabc (Sant_1325-8/Sant_2659-62) |
| Proline | From α-ketoglutarate | — | — | — | PROt2r (Sant_0028)  PROabc (Sant_0944, Sant_0946-7) |
| Lysine | From aspartate | LYSt2r | APC | *SG0955* | LYSt2r (Sant_1419) |
| Asparagine | From aspartate | — | — | — | — |
| Aspartate | From oxaloacetate | ASPt2 | DAACS | *SG2121* | APSt3_3 (Sant_2332)  ASPt2r (Sant_3844)  ASPabc (Sant_2814-7) |
| Isoleucine | From aspartate | ILEt2r | LIVCS | *SG0640* | ILEt2r (Sant_3037) |
| Threonine | From aspartate | — | — | — | — |
| Methionine | From aspartate | METabc | ABC | *SG1915-7* | METabc (Sant_0923-5) |
| Valine | From pyruvate | VALt2r | LIVCS | *SG0640* | VALt2r (Sant_3037) |
| Leucine | From pyruvate | LEUt2r | LIVCS | *SG0640* | LEUt2r (Sant_3037)  LEUabc (Sant_3573-7) |
| Alanine | From pyruvate1 | ALAt2r | AGCS | *SG0408* | ALAt2r (Sant_3406/Sant_2281) |
| Serine | From 3-phospho D-glycerate | SETt2r | HAAAP | *SG0922* | SERt2r (Sant_2673) |
| Cysteine* | From 3-phospho D-glycerate | — | — | — | — |
| Glycine | From 3-phospho D-glycerate | — | — | — | GLYt2r (Sant_3406/Sant_2281) |
| Phenylalanine | From D-erythrose 4-phosphate | PHEt2r | APC | *SG0465* | PHEt2r (Sant_3330) |
| Tryptophan | From D-erythrose 4-phosphate | TRPt2r | APC | *SG0465* | TRPt2r (Sant_3330) |
| Tyrosine | From D-erythrose 4-phosphate | TYRt2r | APC | *SG0465* | TYRt2r (Sant_3330) |
| Histidine | From ribose 5-phosphate | HISt2r | APC | *SG0465* | HISt2r (Sant_3330)  HISabc (Sant_1325-8) |
